# Diagnostic Value of Serum Concentration of Galectin-3 in Patients With Heart Failure With Preserved Ejection Fraction

**DOI:** 10.3389/fcvm.2021.829151

**Published:** 2022-01-24

**Authors:** Jing Jiang, Baojun Yang, Ying Sun, Jing Jin, Zhiying Zhao, Songming Chen

**Affiliations:** ^1^Department of Geriatric Cardiology, Sichuan Provincial People's Hospital, University of Electronic Science and Technology of China, Chengdu, China; ^2^Department of Cardiology, First Affiliated Hospital, Shantou University Medical College, Shantou, China

**Keywords:** galectin-3, diagnosis, diagnostic value, heart failure, heart failure with preserved ejection fraction

## Abstract

**Background:**

Although the predictive value of galectin-3 for heart failure with preserved ejection fraction has been demonstrated, the diagnostic value remains unclear. The present study was performed to address this issue.

**Hypothesis:**

Galectin-3 has diagnostic value for heart failure with preserved ejection fraction.

**Methods:**

This is a diagnostic experiment. We conducted an observational study of 223 patients with combined symptoms of heart failure and diseases that can lead to heart failure with preserved ejection fraction. Patients were grouped into the heart failure group and control group in accordance with the 2016 European Society of Cardiology heart failure guidelines for heart failure with preserved ejection fraction. Baseline information and serum galectin-3 concentration were assessed within 24 h after admission.

**Results:**

Serum galectin-3 concentration was significantly higher in the heart failure group compared with the control group. Binary logistic regression analysis showed that higher galectin-3 concentration was associated with the occurrence of heart failure with preserved ejection fraction. The area under the curve of galectin-3 was 0.763, indicating that galectin-3 has moderate diagnostic value for heart failure with preserved ejection fraction. Galectin-3 >15.974 ng/mL identified heart failure with preserved ejection fraction with 76.0% sensitivity and 71.9% specificity.

**Conclusions:**

There was a correlation between galectin-3 and heart failure with preserved ejection fraction, and galectin-3 was an independent predictor of heart failure with preserved ejection fraction. The diagnostic value of galectin-3 for heart failure with preserved ejection fraction was moderate (AUC: 0.763, 95% CI: 0.696–0.821, *P* < 0.01, and the sensitivity is 76.0% while the specificity is 71.9% at the threshold 15.974 ng/mL) and was higher than that of interventricular septal thickness or E/A ratio.

## Introduction

Heart failure is a well-known and severe cardiovascular syndrome that continues to cause substantial death. According to the 2018 cardiovascular survey report, despite the gradual decline in population mortality rate, heart disease continues to account for a high proportion of deaths, ranking first among all causes of death, and the absolute number will continue to rise (1, 2). Among the different forms of heart failure, heart failure with preserved ejection fraction (HFPEF) has gradually become the most prevalent (3). Recent studies suggested that approximately three-quarters of older adults have HFPEF (4–6). HFPEF was defined as a subform of heart failure by the European Society of Cardiology (ESC) in 2008 and has attracted much attention as it is difficult to diagnose and treat. Epidemiological data show that HFPEF is characterized by high morbidity and mortality (7, 8). A long-term follow-up study of HFPEF suggested that <2% of patients developed heart failure with reduced ejection fraction, and such changes were not associated with mortality in the study population (9). This suggests that HFPEF has a unique pathophysiological basis and requires unique approaches for diagnosis and treatment. The Framingham heart study (10) reported that the rate of mortality from HFPEF is 22–29%, which is slightly lower than that from heart failure with reduced ejection fraction. Kitzman et al. (11) reported that patients suffer from heart failure before left ventricular ejection fraction drops to 50%, and this occurs in all patients with combined systolic and diastolic heart failure. Therefore, it is necessary to identify a reliable method for early identification of HFPEF. Presently, there are many methods for diagnosing HFPEF, such as ultrasonic detection, use of serum B-type natriuretic peptide (BNP) levels, or the combination of multiple diagnostic procedures, which are generally relatively complicated, and with which the specificity and sensitivity cannot be satisfied simultaneously (3, 12). And an early stage of HFpEF can be easily missed (13). We hypothesized that measurement of a biological indicator involved in the pathophysiological pathway of HFPEF can significantly improve the diagnostic efficiency.

Galectin-3 is a soluble β-galactoside-binding lectin secreted by activated cardiac macrophages and is involved in the pathophysiological processes of inflammation and fibrosis (14). It can induce proliferation of cardiac fibroblasts, leading to deposition of collagen in the heart, which can lead to ventricular dysfunction, a process that has been demonstrated in animal studies (15–17). In animal studies, serum galectin-3 level was significantly increased in animal models with volume and (or) stress overload (18), artificially increasing the level of galectin-3 in animals can promote the occurrence of myocardial fibrosis (19). A large number of studies have found that serum galectin-3 level was significantly elevated in either acute or chronic heart failure patients (20). In population studies, patients with higher basal galectin-3 level were more likely to lead to new-onset heart failure (15, 21, 22). And heart failure patients with higher basal galectin-3 level had poorer outcomes (including higher mortality, higher readmission rates, etc.) (23–25). Therefore, galectin-3 has been identified as a prognostic factor for heart failure, especially HFpEF (25–28). The 2013 US guidelines have recommended galectin-3 using for heart failure risk stratification (29), although it has not been used clinically (30–35).

Although the mechanism of action of galectin-3 in the progression of heart failure has been clarified, determining how to use this biomarker still requires investigation. Studies are exploring the diagnostic value of galactose lectin 3 in heart failure, for example: Kanukurti et al. obtained the diagnostic threshold of 10.1 ng/mL, and suggested that galectin-3 and NT-proBNP should be combined for the diagnosis of HFpEF (36); another study mentioned that the cut-off value of 17.8 ng/ml for galectin-3 to diagnose heart failure (37). However, the sample size of the previous studies was small, and the cut-off point value fluctuated greatly. At the same time, mature, stable and cheap assay for galectin-3 has been reported (20). So, what was the reason for galectin-3 still not been used in clinical practice? As far as we concerned, one most important reason was that there is a large discrepancy in current reports regarding diagnostic threshold in heart failure, especially in HFpEF (35–37). Therefore, we conducted the present study to determine the diagnostic value of galectin-3 level for HFPEF and to establish the threshold.

## Materials and Methods

### Study Design and Patient Selection

This is a diagnostic experiment. We conducted an observational study of 223 patients with combined symptoms of heart failure and diseases that can lead to HFPEF such as hypertension, coronary heart disease, and atrial fibrillation. Patients who met these conditions were consecutively admitted to the Department of Cardiovascular Internal Medicine of No.1 Affiliate Hospital of Shantou University Medical College from July 2018 to September 2018.

According to ESC guidelines for heart failure, the upper limit for normal level of BNP in the non-acute setting is 35 pg/mL, while that for N-terminal pro-BNP (NT-proBNP) is 125 pg/mL; in the acute setting, higher values should be used [BNP, 100 pg/mL; NT-proBNP, 300 pg/mL]. The diagnostic value of BNP applies similarly to heart failure with reduced ejection fraction and HFPEF. On average, the values are lower for HFPEF than for heart failure with reduced ejection fraction (38). Echocardiography plays an important role in the diagnosis of heart failure, accounting for two of the four diagnostic criteria for HFPEF. Therefore, we compared the diagnostic value of galectin-3 with that of ultrasonic diagnostic indexes for HFPEF. The diagnostic value of these two indices was significantly higher than that of BNP.

The diagnostic criteria for HFPEF include the following:

1. Typical symptoms of heart failure;
2. Typical signs of heart failure;
3. Small left ventricle with normal or slightly reduced left ventricular ejection fraction (≥50%);
4. The presence of left ventricular structural changes (such as left ventricular hypertrophy or left atrial enlargement) and/or left ventricular diastolic dysfunction. Key functional alterations are an E/e' ratio ≥13.

Patients diagnosed with HFPEF in accordance with the above criteria were eligible for inclusion in the experimental group (HF group). Otherwise, patients were included in the control group. Nineteen subjects with left ventricular ejection fraction <50%, five combined with acute coronary syndrome, and seven who suffered from malignant tumors were excluded. Patients were systematically characterized and clinical data upon admission were recorded in detail. All patients underwent echocardiography upon admission by a skilled echocardiologist. On admission, we assessed several variables, including demographic features, such as age, sex, history of hypertension (including duration of hypertension and pressure level), diabetes, coronary artery disease, atrial fibrillation, New York Heart Association (NYHA) basal functional status, and history of drug therapy. All patients signed a written consent form and the Ethics Investigation Committee approved the study.

### Serum Concentration of Galectin-3 and Echocardiography

Blood samples were collected on the day subjects underwent echocardiography. Collected supernatants were centrifuged at 3,000 rpm for 15 min at 4°C. Serum concentration of galectin-3 was measured using an enzyme-linked immunosorbent assay protocol without cross-reactivity with collagens or other galectins. Detailed echocardiography was performed to evaluate parameters of diastolic function in accordance with published recommendations and guidelines (39).

The objective of this analysis was to compare the diagnostic value of galectin-3 in HFPEF with the relatively common echocardiographic criteria of HFPEF.

### Statistical Analysis

Baseline features were compared between the two groups (HF group and control group). Continuous variables were tested for normal distribution using the Kolmogorov–Smirnov test. Results are presented as mean (standard deviation, SD) for continuous variables, and as number (%) for categorical variables. Receiver operating characteristic (ROC) curves were established, and the area under the ROC curves (AUC) was calculated. AUCs were compared using the DeLong test. Sensitivity and specificity above the third and highest point of the Youlden index were used as indicators. We supplemented the analyses of diagnostic capacity by adjusting for age, sex, renal function, diabetes, hypertension, and atrial fibrillation. All statistical analyses were performed using SPSS software (version 25.0, Chicago, IL, USA). A two-sided *P* value < 0.05 was considered statistically significant.

## Results

### Baseline Features

In accordance with the 2016 ESC guidelines for heart failure, 192 patients were enrolled. Among them, 96 met the criteria of HFPEF and were included in the HF group, while the remaining 96 were included in the control group. Baseline features are shown in Table 1. There were no significant differences in baseline features between the two groups. Overall, mean age was 66 years (interquartile range, 15.00), and 47.9% were female. Comorbidities included hypertension (81.8%), coronary heart disease (33.9%), diabetes (29.7%), and atrial fibrillation (8.3%). Mean concentration of serum galectin-3 was 16.33 ng/mL (SD, 3.504). Serum concentration of galectin-3 was significantly higher in the HF group compared with the control group (*P* < 0.001). Regarding echocardiogram data, the interventricular septal thickness, left ventricular posterior wall thickness, left atrial diameter, E peak, E/A ratio, and E/e ratio were higher in patients in the HF group, whereas left ventricular ejection fraction and e peak were lower in this group.

**Table 1:** Baseline demographic and clinical features of the study population.

**Variables**	**All subjects (*n* = 192)**	**HF group (*n* = 96)**	**Control group (*n* = 96)**	**Statistical value**
Female	92 (47.9%)	52 (54.2%)	40 (41.7%)	0.112
Age, y	66.00 (15.000)	69.00 (19.000)	63.00 (14.000)	0.062
Medical history				
CHD	65 (33.9%)	33 (34.4%)	32 (33.3%)	1.000
Hypertension	157 (81.8%)	75 (78.1%)	82 (85.4%)	0.262
Blood pressure level				0.694
Level 1	23 (12.0%)	8 (8.3%)	15 (15.6%)	
Level 2	48 (25.0%)	26 (27.1%)	22 (22.9%)	
Level 3	86 (44.8%)	41 (42.7%)	45 (46.9%)	
Time of HBP, y	4.00 (9.000)	3.00 (9.000)	4.00 (9.000)	0.803
Diabetes,	57 (29.7%)	31 (32.3%)	26 (27.1)	0.528
Atrial fibrillation	16 (8.3%)	11 (11.5%)	5 (5.2%)	0.190
NYHA classification				0.070
NYHAI	107 (55.7%)	48 (50.0%)	59 (61.5%)	
NYHAII	69 (35.9%)	37 (38.5%)	32 (33.3%)	
NYHA III	11 (5.7%)	7 (7.3%)	4 (4.2%)	
NYHA IV	5 (2.6%)	4 (4.2%)	1 (1.0%)	
Drug therapy history				
No. of drugs	2.00 (1.000)	2.00 (2.000)	2.00 (1.000)	0.437
ACEI,	59 (30.7%)	29 (30.2%)	30 (31.3%)	1.000
ARB	43 (22.4%)	23 (24.0%)	20 (20.8%)	0.729
CCB	98 (51.0%)	48 (50.0%)	50 (52.1%)	0.885
β-Blocker	79 (41.1%)	42 (43.8%)	37 (38.5%)	0.558
DU	48 (25.0%)	28 (29.2%)	20 (20.8%)	0.243
Data of ECG				
IVS, mm	12.00 (3.000)	12.00 (2.000)	11.00 (3.000)	0.007
LVPW, mm	11.00 (3.000)	11.00 (3.000)	11.00 (2.000)	0.004
LVD, mm	45.00 (7.000)	45.00 (7.000)	44.00 (7.000)	0.184
LA, mm	30.00 (5.00)	31.00 (7.000)	29.00 (5.000)	0.001
LVEF, %	67.00 (7.00)	67.00 (8.000)	67.00 (6.000)	0.133
E peak	73.5 (29.000)	79.00 (28.000)	66.00 (27.000)	0.000
A peak	83.84 ± 20.305	83.52 ± 20.479	84.21 ± 20.211	0.815
e peak	6.00 (4.000)	5.00 (2.000)	8.00 (3.000)	0.000
a peak	11.00 (4.000)	11.00 (5.000)	11.00 (3.000)	0.812
E/A ratio	0.85 (0.517)	0.89 (0.548)	0.77 (0.466)	0.000
E/e ratio	15.08 (7.591)	16.25 (2.100)	8.67 (3.191)	0.000
Galectin-3, ng/ml	16.33 ± 3.504	17.90 ± 3.458	14.51 ± 2.569	0.000

*Data are presented as number (%) for categorical variables, and as mean ± SD for continuous variables that conformed to a normal distribution. Other data that did not conform to a normal distribution are presented as median (IQR). Groups were compared using Chi-square, ANOVA, or Kruskal –Wallis tests. ECG, echocardiography; CHD, coronary heart disease; HBP, hypertension; NYHA, New York Heart Association; ACEI, Angiotensin-converting enzyme inhibitor; ARB, Angiotensin receptor blocker; CCB, Calcium channel blocker; B, Beta-adrenergic receptor blocker; DU, Diuretic; IVS, interventricular septal thickness; LVPW, left ventricular posterior wall thickness; LVD, Left ventricular diastolic diameter; LA, Left atrial diameter; LVEF, Left ventricular ejection fraction; E peak, Early diastolic mitral inflow peak velocity; A peak, late diastolic mitral inflow peak velocity; e peak, early diastolic Doppler spectrum of mitral valve; a peak, late diastolic Doppler spectrum of mitral valve*.

### Binary Logistic Regression Analysis

To identify risk factors for HFPEF, we analyzed variables in which differences were observed between the HF and control groups but had no correlation between each other in regression analysis. The results showed that increased galectin-3 concentration increased the risk of HFPEF by approximately 57.4% [odds ratio (OR) 1.574, 95%CI: 1.368, 1.812, *P* < 0.01]. Interventricular septal thickness and E/A ratio also significantly increased the risk of HFPEF, OR and 95%CI were 1.571, (1.247, 1.979); 4.938, (1.634, 14.918), respectively, *p* values were all <0.01 (see Table 2).

**Table 2:** Logistic regression analysis.

	**B value**	**Wald**	***P* value**	**OR value**	**95% C.I. of OR value**	
					**Lower limit**	**Upper limit**
LVEF	−0.041	1.132	0.287	0.960	0.891	1.035
Galectin-3	0.454	40.096	<0.01^**^	1.574	1.368	1.812
LA	0.086	3.057	0.080	1.090	0.990	1.200
IVS	0.452	14.697	<0.01^**^	1.571	1.247	1.979
E/A value	1.597	8.014	0.005^**^	4.938	1.634	14.918

*IVS, interventricular septal thickness; LA, left atrial diameter; LVEF, left ventricular ejection fraction*.

** *The difference was statistically significant*.

### ROC Curve and AUC Analysis

**Figure 1:**
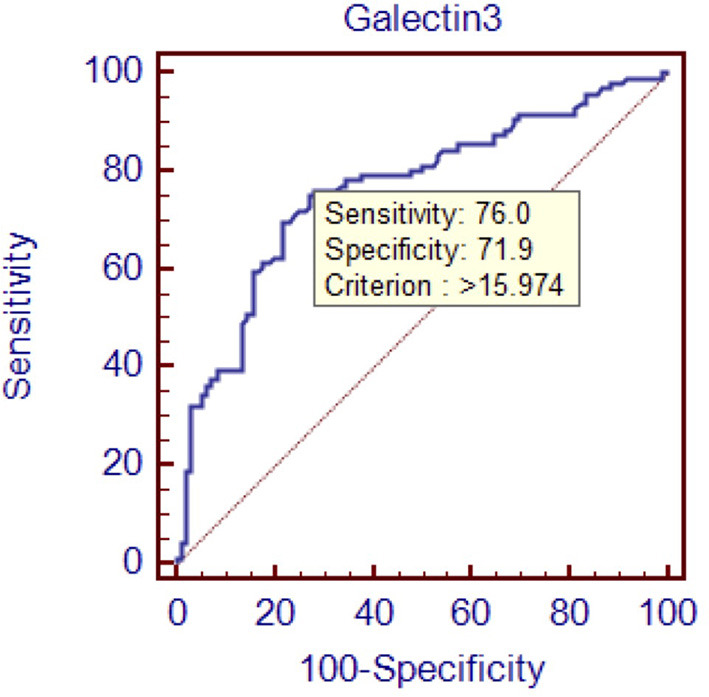
Receiver operating characteristic curve of galectin-3 to diagnose HFPEF. Area under the curve: 0.763, 95% C.I. (0.696–0.821), *P* < 0.0001.

**Figure 2:**
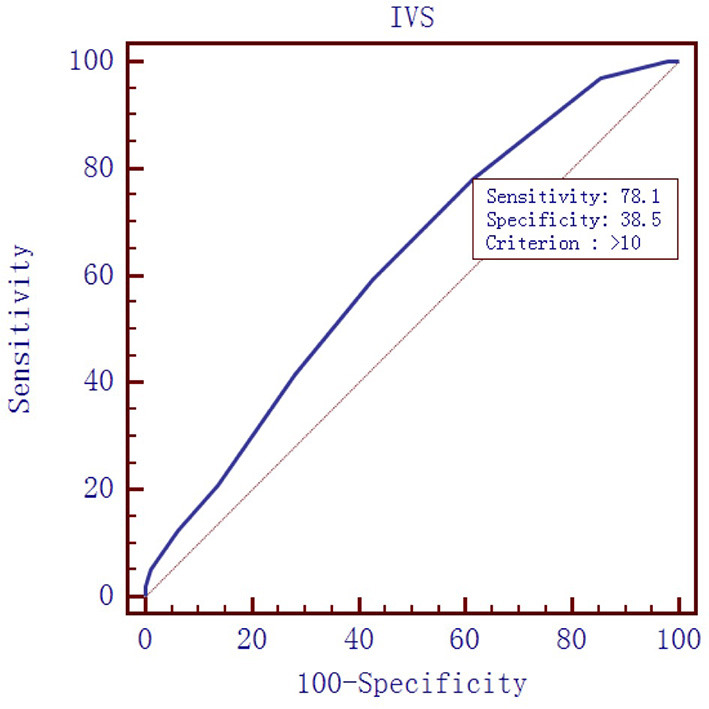
Receiver operating characteristic curve of interventricular septal (IVS) thickness to diagnose HFPEF. Area under the curve: 0.619, 95% C.I. (0.546–0.688), *P* = 0.0028.

**Figure 3:**
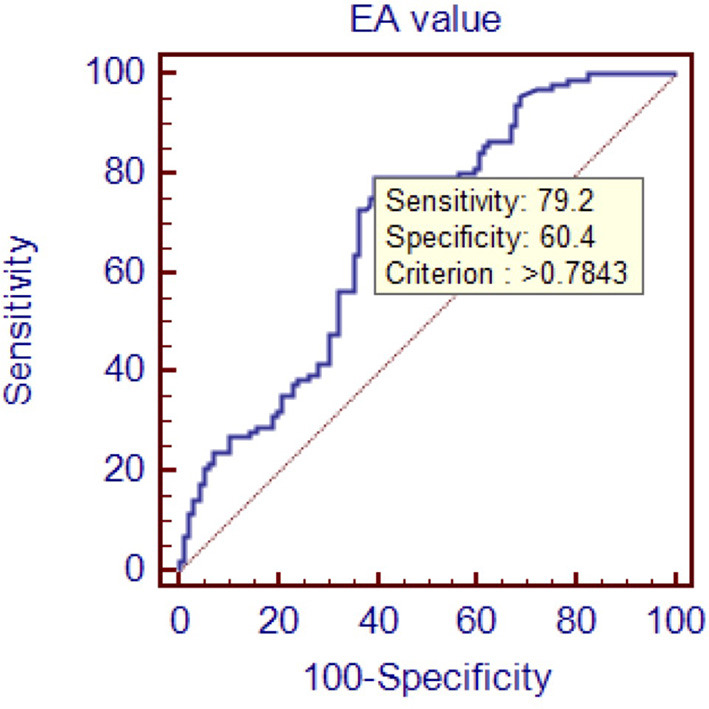
Receiver operating characteristic curve of E/A value to diagnose HFPEF. Area under the curve: 0.688, 95% C.I. (0.618–0.753), *P* < 0.0001.

**Figure 4:**
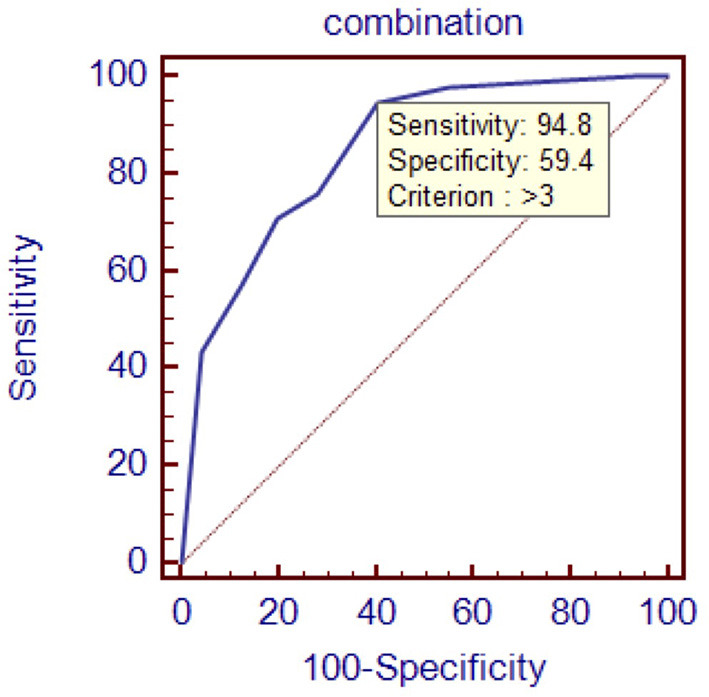
Receiver operating characteristic curve of galectin-3 combined with interventricular septal thickness and E/A value. Area under the curve: 0.850, 95% C.I. (0.792–0.898), *P* < 0.0001.

To evaluate the diagnostic value of galectin-3 level for HFPEF, an ROC curve was constructed using MedCal software based on galectin-3 concentration. The AUC was 0.763 (95% confidence interval (C.I.), 0.696–0.821, *P* < 0.01), and galectin-3 >15.974 ng/mL identified HFPEF with 76.0% sensitivity and 71.9% specificity. The AUC rose to 0.850 when galectin-3 was combined with interventricular septal thickness and E/A value, while the sensitivity rose to 94.8%. ROC analyses are shown in Figures 1–4, and data pertaining to the ROCs are shown in Table 3.

**Table 3:** ROC data.

**variables**	**Sensitivity (%)**	**Specificity (%)**	**Cutoff point**	**AUC**
Galectin-3	76	71.9	15.974 ng/ml	0.763
IVS	78.1	38.5	10 mm	0.619
E/A	79.2	60.4	0.7843	0.688
Combination	94.8	59.4	/	0.850

*IVS, interventricular septal thickness; E peak, Early diastolic mitral inflow peak velocity; A peak, late diastolic mitral inflow peak velocity; AUC, Area under curve*.

## Discussion

In the present study, we found that galectin-3 secretion was significantly increased in patients with HFPEF. This observation is consistent with recent findings (15–17, 40). In a study of 119 subjects, Gopal et al. demonstrated that galectin-3 level was increased in patients with heart failure, regardless of type (41). One study found that for each 1-ng/mL increase in galectin-3 concentration, the rate of heart failure readmission increased by 18% (35). Another study found that the risk of heart failure increased by 28% for each SD increase in galectin-3 concentration (26). Recent studies suggest that the targets of galectin-3 in myocardial fibroblasts and extracellular matrix, together with activated myocardial macrophages, can induce fibroblast activation and proliferation, stimulate infiltration of macrophages and mast cells, increase myocardial interstitial deposition of molecules such as type I collagen around the heart and blood vessels, and cause myocardial hypertrophy and decreased myocardial compliance, which ultimately lead to heart failure (42). Therefore, galectin-3 concentration in patients with HFPEF is higher than that in patients with heart failure without preserved ejection fraction, which confirms the microscopic mechanism from a macro perspective.

Binary logistic regression analysis showed that higher galectin-3 concentration was associated with the occurrence of HFPEF. Therefore, galectin-3 is an independent risk factor for HFPEF and can predict its occurrence. In this study, binary logistic regression analysis of galectin-3 concentration and HFPEF was consistent with previous studies and confirmed the value of using galectin-3 concentration for predicting HFPEF. Other studies made similar observations (20, 21, 23–26, 35, 40, 43–46), and galectin-3 has been approved for prognostic use in heart failure in the United States (29).

In fact, galectin-3 has proven to be predictive of morbidity and poor prognosis in many other diseases, not just heart failure. In diabetes, galectin-3 may mediate b-cell fibrosis through an inflammatory pathway, leading to impaired insulin secretion (47). In patients with cirrhosis, hepatocytes were induced to secrete Galectin-3, while normal hepatocytes had a decreased scavenging effect on galectin-3, which ultimately leaded to an increase in serum galectin-3 concentration, while the latter promoted the deterioration of liver cirrhosis by inducing activation of hepatic stellate cells and fiber synthesis (48). High levels of galectin-3 was thought to be associated with renal interstitial fibrosis, renal tubule atrophy, and endovascular fibrosis, possibly through immune reaction-related pathways (49). Elevated galectin-3 level was associated with the development of heart failure in hypertension patients (19). In galectin-3 knockout mice, a high-fat diet did not cause fat cell hypertrophy, suggesting that galectin-3 was involved in the pathogenesis of obesity (50). Galectin-3 was also demonstrated to be an independent predictor of all-cause and cardiovascular death in patients with systemic sclerosis (51). There were no significant differences in baseline data in our study, and renal function, BMI and other data were not matched. However, a high-quality, large-sample study showed that galectin-3 was an independent predictor of mortality, even after adjusting for factors such as blood pressure, lipids, kidney function, and BNP (14). Therefore, our results remain highly reliable.

To determine the diagnostic value of galectin-3 for HFPEF, an ROC curve was constructed and the AUC was calculated. The AUC was 0.763, indicating that galectin-3 has moderate diagnostic value for HFPEF. At the cutoff point of 15.974 ng/mL, the diagnostic sensitivity and specificity of galectin-3 for HFPEF was 76.0 and 71.9%, respectively, and were significantly higher than those of the other two risk factors (interventricular septal thickness and E/A value). There are prior reports on the diagnostic capacity of galectin-3 for heart failure, although most of these involved comparisons with BNP and other indicators. Javier Carrasco-Sánchez et al. obtained an AUC of 0.630 for galectin-3, and although this result was not ideal, it was superior to that of NT-proBNP (33). In our study, the diagnostic value of galectin-3 was compared with the current “gold standard” for heart function diagnosis in accordance with the latest heart failure diagnostic guidelines. Additionally, we found that galectin-3 had a higher diagnostic value than interventricular septal thickness and E/A ratio, two indicators that are clearly associated with E/E, which further demonstrated the high value of galectin-3 for diagnosing HFPEF. There were differences in cutoff points between our study and others. In the study by Chen et al., the cutoff point was 7.52 ng/mL (44), whereas Trippel et al. selected 17.8 ng/mL as the cutoff point (35). The large differences in cutoff points may be related to the study populations, sampling times, and testing methods. For example, in the study by Chen et al. the participants had chronic heart failure, while ours had HFPEF, and the ratio of NYHA III–IV was much higher than in our study (44).

A long-term follow-up study showed a significant increase in the rate of new-set heart failure with increased galectin-3 concentrations (21), Another spanning 10 years study also found that dynamically elevated galectin-3 level was more closely associated with new-set heart failure and mortality (52). This phenomenon can be explained by cellular mechanisms. Cardiac failure with fractional ejection retention was characterized by increased myocardial stiffness and myocardial interstitial fibers (53). An *in vitro* cell experiment showed increased secretion of galectin-3 after stretching cardiac muscle cells (18), Galectin-3 can increase infiltration of macrophages and mast cells in myocardial cells, leading to myocardial fibrosis, myocardial stiffness and left ventricular dysfunction (15, 19). A series of previous studies have confirmed a causal relationship between galectin-3 and cardiac remodeling (21). This explained that why there was diagnostic value of Galectin-3 in HFpEF.

The early diagnosis of HFpEF was the shortcoming of heart failure diagnosis at present (13), the diagnostic significance of this stage was to early manage and reduce the incidence of subsequent acute heart failure or other symptomatic heart failure (such as heart failure in stage C or D), and to reduce the depletion of medical resources. Studies had concluded that in the diagnosis of heart failure, because BNP is more sensitive to volume overload, galectin-3 is more sensitive to fibrosis (37), a combination of the two was recommended to increase diagnostic accuracy (36, 37). Such advice had some merit, but was bound to increase healthcare costs. Echocardiography for the diagnosis of heart failure with retained ejection fraction has been the basis of current diagnosis, but the examination process of echocardiography was time-consuming, relatively expensive, and subject to subjective influence by personnel. One report suggests that galectin-3 may be an alternative to ultrasound in diagnosing diastolic dysfunction (54). Galectin-3 showed considerable sensitivity and specificity, with low economic cost, time cost and labor cost. Therefore, galectin-3 may be considered for the diagnosis of HFpEF alone.

This study had some limitations. First, the sample size was relatively small. Future studies in larger populations are necessary to further clarify the diagnostic value of galectin-3. Second, we used the cardiac ultrasound E/e ratio as the gold standard reference for diagnosis, and although the values were measured by experienced echocardiographers, they were not performed by the same individual. Therefore, there may have been errors in the measured values because of the subjectivity of ultrasound diagnosis.

## Conclusions

In this study, increased galectin-3 level was observed in patients with HFPEF. Galectin-3 level was demonstrated to be a risk factor for HFPEF (AUC: 0.763, 95% CI: 0.696–0.821, *P* < 0.01, and the sensitivity is 76.0% while the specificity is 71.9% at the threshold 15.974 ng/mL). Galectin-3 has moderate diagnostic value for HFPEF. Owing to the overall lack of evidence in this area, more studies are necessary to verify our conclusions.

## Data Availability Statement

The raw data supporting the conclusions of this article will be made available by the authors, without undue reservation.

## Ethics Statement

The studies involving human participants were reviewed and approved by Ethics Committee of First Affiliated Hospital of Shantou University Medical College. The patients/participants provided their written informed consent to participate in this study.

## Author Contributions

All authors listed have made a substantial, direct, and intellectual contribution to the work and approved it for publication.

## Funding

This study was supported by Health Research Project for Cadres of Sichuan Province (No. 2020-202) and Key R&D Project of Science and Technology Department of Sichuan Province (No. 2020YFSY0045).

## Conflict of Interest

The authors declare that the research was conducted in the absence of any commercial or financial relationships that could be construed as a potential conflict of interest.

## Publisher's Note

All claims expressed in this article are solely those of the authors and do not necessarily represent those of their affiliated organizations, or those of the publisher, the editors and the reviewers. Any product that may be evaluated in this article, or claim that may be made by its manufacturer, is not guaranteed or endorsed by the publisher.
